# The relation between project team conflict and user resistance in software projects

**DOI:** 10.1371/journal.pone.0260059

**Published:** 2021-11-16

**Authors:** Simon Vrhovec, Blaž Markelj

**Affiliations:** Faculty of Criminal Justice and Security, University of Maribor, Ljubljana, Slovenia; Universiti Pertahanan Nasional Malaysia, MALAYSIA

## Abstract

This study aims to explore the relation between conflict in the project team and user resistance to change in software projects. Following a cross-sectional research design, a survey was conducted among 1,000 largest companies in Slovenia (N = 114). The results of PLS-SEM analysis indicate that task and process conflicts in the project team are associated with user resistance. This study is among the first to associate conflict within the project team and user resistance in the implementing organization. It is also one of the first studies to investigate the relations between different types of conflict and user resistance. Project managers may invest resources into adequately managing conflicts within the project team related to tasks in which the project team interacts with users of developed software to lower user resistance. Project with poorly defined roles (e.g., agile and information security projects) may be more prone to user resistance than projects with clearly defined roles.

## 1 Introduction

Organizations are continuously investing in information technology for improved productivity and innovation gains [1]. The gains of new software are dependent on the implementation success and adoption by key stakeholders [1, 2]. Resistance to change, sometimes referred to more specifically as user resistance or more generally as stakeholder resistance [3, 4], is among the most significant barriers to the adoption of new software in various contexts, such as healthcare [4–7], government services [8], supply chain [9], human resources [10], decision support [11], knowledge sharing [12], information security [13, 14], IT services [15] and software development [16], and so forth.

Resistance to change has been extensively researched for over seven decades aiming to explain why and how individuals resist change, including information systems and software implementations [2, 3]. Even though it has a negative connotation, resistance is neither negative nor positive by itself and can contribute to better project outcomes [3]. The causes of resistance to change have been linked to organizational, social, political, technical and contextual factors [4]. Antecedents of resistance to change are related to various stakeholders (e.g., users, management, customers) [3].

In this study, we focus on user resistance in software projects. Several antecedents of user resistance were identified in information systems research. For example, user resistance may be related to users’ perceptions about the new technology and about the impact of new technology on their work routines [2, 17]. Similarly, user resistance has been associated with the costs associated with switching to the new way of working [1, 18]. The published literature also suggests that various kinds of perceived threats to an individual (e.g., perceived loss of professional autonomy, perceived dissatisfaction, threats to status, knowledge) also relate to user resistance [1, 3, 4, 17]. Resistance to change is however not only about work as social and power relations are also important in organizations. For example, user resistance may be associated with social influence and norms [1, 18], authoritarian leadership style [19], top management support [20] and struggle for power [3]. Although most research on user resistance focused on factors directly associated with users and their organizational context as presented, user resistance has been also associated with issues related to the change implementation itself (e.g., communication issues, user involvement) [3, 17, 21, 22]. Nevertheless, research on the associations between matters going on within the project team developing new software and user resistance seems particularly scarce as, to the best of our knowledge, has not been directly investigated before.

When individuals with varying values work together, conflict is practically unavoidable [23]. Conflict can be categorized into three key types: relationship, task and process conflict [24–26]. *Relationship conflict* refers to disagreements and incompatibilities between individuals regarding personal issues that are not related to tasks [23]. *Task conflict* refers to differences in opinions, viewpoints and ideas about how tasks should be performed [26, 27]. *Process conflict* refer to varying opinions about how tasks are successfully achieved (e.g., responsibilities and resource delegation) [24, 26]. Like resistance to change, studies show that conflict can contribute to better project outcomes [28]. While relationship conflict is generally associated with negative consequences [29], task and process conflicts may be associated with both negative and positive outcomes depending on how they are handled [28–30]. Task and process conflicts are also commonly referred to as constructive conflict [28, 29].

Team conflict has been well-researched in information systems research. For example, conflict has been studied in the context of software development process and software projects [26, 27, 29, 31–33], employee well-being [23, 25], creativity and innovation [28, 34], organizational and team performance [30, 35]. Literature on resistance and team conflict has been however developed separately with few studies attempting to merge knowledge from both streams of research [36]. Although the relationship between user resistance and conflict in the implementing organization has been investigated before [4, 19, 36–38], it remains unclear whether conflict within a project team is related to user resistance in the implementing organization.

The primary objective of this study is to investigate how is project team conflict related to user resistance in software projects thus addressing the above presented gaps in our knowledge. This paper makes two key contributions to the literature. First, this is one of the first studies to investigate the impact of conflict within software project teams on resistance to project outcomes thus contributing to the literature on resistance to change. Second, this study further contributes to the resistance to change literature by being among the first studies to directly associate different types of conflict within software project teams with user resistance in the implementing organization.

## 2 Research model

In this study, we propose and empirically test a research model comprising of three hypotheses. User resistance has been related to various conflicting interests between stakeholders in the implementing organization [3, 4, 39]. Project teams however often involve members external to the implementing organization (e.g., software development is outsourced to software companies). Nevertheless, matters internal to the project team may affect the user resistance in the implementing organization even if the project is fully outsourced as it may affect antecedents of user resistance, such as communication and user involvement [3, 17, 21, 22]. Since all three types of conflicts can affect team performance [24, 30, 35], including the performance of the interaction with users of developed software, we posit the following hypotheses:

*H1*: Relationship conflict is positively associated with user resistance to change.
*H2*: Task conflict is positively associated with user resistance to change.
*H3*: Process conflict is positively associated with user resistance to change.

## 3 Method

### 3.1 Design

A cross-sectional survey research design was used to explore the associations between conflict and resistance to change related to software projects.

### 3.2 Ethical considerations

This study did not require an approval from the Institutional Review Board according to the legislation of the Republic of Slovenia and internal acts of the University of Maribor.

### 3.3 Measures

Theoretical constructs were defined and operationalized as presented in Table 1. All measured constructs were reflective, and their items were adapted from previously validated items. Items for *relationship conflict*, *task conflict* and *process conflict* were adapted from [24]. Items for *resistance to change* were adapted from [40]. All items were measured by using a 7-point Likert scale from 1 “strongly disagree” to 5 “strongly agree”.

**Table 1:** Definitions of theoretical constructs.

Theoretical construct	Operational definition
Relationship conflict [RC]	The extent of relationship conflict in a software project.
Task conflict [TC]	The extent of task conflict in a software project.
Process conflict [PC]	The extent of process conflict in a software project.
Resistance to change [RtC]	The extent of to which resistance to change manifested due to a software project.

The survey was distributed in Slovenian which was the primary language of all respondents in our study. All items were developed by following a predefined protocol as follows. The questionnaire was first developed in English and then translated into Slovenian. To ensure the consistency between the Slovenian and English questionnaire, the Slovenian questionnaire was translated back to English. No significant differences in the meaning between the original items in English and back-translations were noticed.

### 3.4 Sample and data collection

We conducted a survey among the heads of IT departments (i.e., unit of observation). The heads of IT departments have an insight into IT projects, including software projects, conducted at their companies. We first asked them to choose an important software project that has finished in the last three years and that they remember well (i.e., unit of analysis). Heads of IT departments were then asked to complete the survey about the chosen project.

The population of the web survey consisted of heads of IT departments in 1,000 largest companies in Slovenia. A total of 117 respondents completed the survey providing for a response rate of 11.7 percent. After excluding poorly completed responses, we were left with *N* = 114 useful responses for further analysis. The data were gathered from February to June 2016.

Table 2 provides an overview of the project sample characteristics.

**Table 2:** Sample characteristics.

	Frequency	Percent
**Software development methodology**		
Agile methodology	9	7.9
Traditional methodology	37	32.5
No formal methodology	68	59.6
**Software type**		
New custom software solution	32	28.1
Customized standard software solution from a local provider	39	34.2
Customized standard software solution from an international provider	42	36.8
*N/A*	1	0.9
**Project team size**		
Up to 5 team members	28	24.6
6–10 team members	46	40.4
11–15 team members	12	10.5
16–20 team members	9	7.9
21–25 team members	2	1.8
26 and more team members	11	9.6
*N/A*	6	5.3
**Project costs**		
Less than 100,000 €	67	58.8
Between 100,000 € and 250,000 €	21	18.4
Between 250,000 € and 500,000 €	12	10.5
Between 500,000 € and 1,000,000 €	4	3.5
Between 1,000,000 € and 2,500,000 €	4	3.5
Between 2,500,000 € and 5,000,000 €	3	2.6
Between 5,000,000 € and 10,000,000 €	1	0.9
10,000,000 € or more	1	0.9
*N/A*	1	0.9
**Project duration**		
Less than 3 months	17	14.9
Between 3 and 6 months	30	26.3
Between 6 and 12 months	33	28.9
Between 12 and 18 months	11	9.6
Between 18 and 24 months	7	6.1
Between 24 and 30 months	3	2.6
36 months or more	3	2.6
*N/A*	10	8.8

### 3.5 Data analysis

Consistent partial least squares structural equation modeling (PLSc-SEM) was used to test the proposed research model. PLSc-SEM is suitable for analyzing data when the sample size is small [41] as is the case in our study. The collected data were processed with IBM SPSS Statistics 28 (descriptive statistics only), R version 3.6.3, and cSEM version 0.4.0 [42].

There were 0.9 percent missing values which were imputed with medians before data analysis with PLSc-SEM. The survey instrument was first validated with a confirmatory factor analysis. Construct items were tested for their reliability, convergent validity and discriminant validity. Reliability was tested by calculating composite reliability (*CR*). Values above 0.60 are acceptable and values above 0.70 are recommended [43]. Convergent validity was determined examining average variance extracted (*AVE*). Values above 0.50 are recommended however values below this threshold may be acceptable if *CR* is adequate [43, 44]. Discriminant validity was determined by heterotrait-monotrait ratio of correlations (HTMT) analysis.

A structural model was then constructed to test the hypotheses. The structural models were estimated with bootstrap resampling with 5,000 replications.

## 4 Results

### 4.1 Instrument validation

A measurement model was developed to validate the survey instrument. Table 3 presents *CR*, *AVE* and HTMT analysis which are relevant for determining the validity and reliability of the survey instrument. First, *CR* ranged from 0.732 to 0.907 thus exceeding the commonly accepted threshold 0.70. This demonstrates adequate reliability of all constructs. Next, *AVE* ranged from 0.549 to 0.766. Values above the 0.50 threshold are generally considered as adequate therefore indicating adequate convergent validity. Additionally, factor loadings (see Table 4) except for TC1 were above the 0.70. A low factor loading for TC1 may be an indication that this item may not measure the corresponding theoretical construct well. After carefully considering the other items (i.e., TC2 and TC3) and comparing our results to the results in published literature (e.g., [24]), we found no obvious reasons for a low factor loading. Explanations for the divergence may thus need to be sought elsewhere. For example, published literature measured task conflict for on-going group activities but our study measured task conflict for a recently completed project. It is possible that respondents better or more strongly remember conflicts of ideas (TC1, *M*_*TC*1_ = 3.50) than disagreements about tasks (TC2, *M*_*TC*2_ = 2.78) or conflicting opinions about the project (TC3, *M*_*TC*3_ = 3.04). Since all other indicators suggested adequate convergent validity, we nevertheless did not consider this as a serious issue. Finally, HTMT ratios of correlations were all below the conservative 0.85 threshold thus indicating adequate discriminant validity of the survey instrument.

**Table 3:** Validity and reliability of the survey instrument. Composite reliability (*CR*), average variance extracted (*AVE*), and heterotrait-monotrait ratio of correlations (HTMT) analysis.

Construct	*CR*	*AVE*	1	2	3
1. RC	0.891	0.732			
2. TC	0.759	0.549	0.622		
3. PC	0.907	0.766	0.801	0.601	
4. RtC	0.732	0.577	0.613	0.616	0.684

*Notes*: RC—relationship conflict; TC—task conflict; PC—process conflict; RtC—resistance to change.

**Table 4:** Questionnaire items.

Construct	Loading	Item	Source
Relationship conflict	0.873	RC1. There has been much relationship tension in the project team.	[24]
	0.908	RC2. People often got angry while working in the project team.	[24]
	0.780	RC3. There has been much emotional conflict in the project team.	[24]
Task conflict	0.301	TC1. There has been much conflict of ideas in the project team.	[24]
	0.862	TC2. There have been frequent disagreements within the project team about the task of the project.	[24]
	0.902	TC3. People in the project team frequently had conflicting opinions about the project.	[24]
Process conflict	0.850	PC1. There have been frequent disagreements about who should do what in the project team.	[24]
	0.957	PC2. There have been many conflicts about task responsibilities in the project team.	[24]
	0.812	PC3. There have been frequent disagreements about resource allocation in the project team.	[24]
Resistance to change	0.774	RtC1. There have been many users resisting the project or the deployed solution.	[40]
	0.746	RtC2. There have been many cases in which user departments did not reply to the request of the project team.	[40]

To reduce the likelihood of social desirability bias, we informed the respondents that participation in the research is voluntary and anonymous.

### 4.2 Structural model

**Fig 1:**
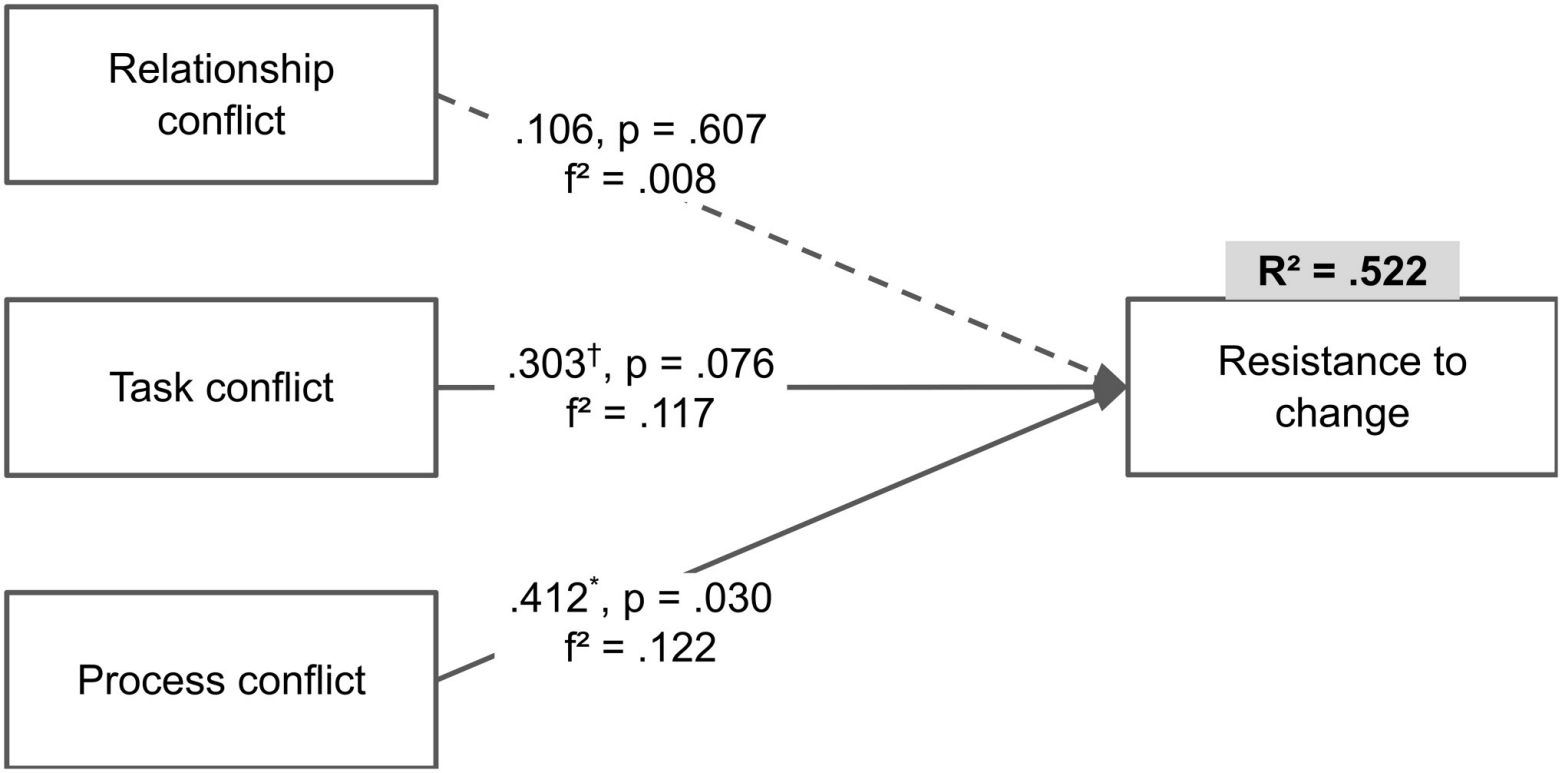
Structural model. *Notes*: ^†^ *p* < 0.10, *p < 0.05.

A structural model was developed to test the hypotheses. Fig 1 presents standardized path coefficients, their *p*-values, effect sizes *f*^2^, and adjusted *R*^2^. Predictor constructs (i.e., relationship conflict, task conflict and process conflict) explain a meaningful share of variance of the predicted construct (i.e., resistance to change) thus indicating that the results are meaningful.

The results support hypotheses H2 (*p* = 0.076) and H3 (*p* = 0.030). Nevertheless, both associations have small effect sizes (i.e., *f*^2^ < 0.15). The results however do not support hypothesis H1 (*p* > 0.10).

The summary of hypotheses testing is presented in Table 5.

**Table 5:** Hypotheses testing summary.

Hypothesis		Evidence	Conclusion
H1	Relationship conflict is positively associated with user resistance to change.	Non-significant positive path	Not supported
H2	Task conflict is positively associated with user resistance to change.	Significant positive path, small effect size	Supported
H3	Process conflict is positively associated with user resistance to change.	Significant positive path, small effect size	Supported

## 5 Discussion

The purpose of this study was to investigate how is project team conflict related to user resistance in software projects. The results of our study suggest several theoretical and practical implications as discussed in the following subsections.

### 5.1 Theoretical implications

This study has several theoretical implications. First, this study provides evidence that conflict within the project team are related to user resistance in the implementing organization. This contributes to the literature on resistance to change by broadening the sources of user resistance due to conflict beyond the conflict between stakeholders in the implementing organization (e.g., struggle for power) [3, 4, 39]. Although users are not directly involved in project team conflicts and may not be even aware of them, the results of our study indicate that the consequences of those conflicts may affect them nevertheless. Although we employed a cross-sectional research design, the nature of the studied phenomena suggests a causal relation between project team conflict and user resistance. User resistance can occur at any stage of the implementation (i.e., pre-implementation, during implementation and post-implementation) [3] and may therefore precede the start of the project and the formation of the project team. It may be nevertheless more likely that resistance to change was caused by poor communication or disappointing user involvement due to conflict in the project team than conflict within the project team caused by user resistance. User resistance may not emerge during a software project, and its team members may not be aware of it even if it would or may simply underestimate its extent until late in a project [20].

Second, the results of our study suggest that only constructive (i.e., task and process [28]) conflict is associated with resistance to change. The absence of a significant association between relationship conflict and user resistance to change may offer additional support for assuming a causal relationship between conflicts and resistance since constructive conflict is directly related to project tasks, including tasks in which the project team interacts with users. These activities, such as activity on social media, are related to user resistance [21]. Since resistance to change is a symptom resulting from an underlying root cause [3], a positive relationship between constructive conflict and resistance to change indicates that constructive conflict was probably not managed adequately in our sample. Improving conflict management may therefore help to tackle resistance to change. This is analogue to existing research suggesting that adequate conflict management in the implementing organization can result in decreased user resistance [19].

### 5.2 Practical implications

This study also has some practical implications for different stakeholders. First, project managers should focus on management of conflict within the project team related to tasks in which the project team interacts with users to lower the probability of manifestation of user resistance. This study may offer project managers a new perspective on the priorities for conflict management. Although management of conflicts related to software development is important, project managers need to keep an eye on “auxiliary” tasks (such as communication activities and user involvement) too since they may significantly affect the overall project success beyond the iron triangle (i.e., time, cost and quality).

Second, software projects with poorly defined, unclear or ambiguous roles (e.g., agile software development and information security, especially in small and medium enterprises without information security departments) may be more prone to user resistance than projects in which roles are clearly defined. For example, roles in information security are complex and may fuel process conflicts in software projects which include elements of information security [45]. With its rising importance for organizations, information security is becoming embedded in more and more software projects and often conflicts with the way employees have done their job for years [46].

### 5.3 Limitations and future research

Some limitations need to be considered when interpreting the results of this study. First, the study was conducted in a single country. The findings may not be applicable across different countries and cultural contexts. Future work in in other countries would be helpful to address this limitation and broaden the ecological validity of this study. Second, the findings of this study may not be generalized to all types of companies by size, especially the small and micro companies since it was conducted among the 1,000 largest companies in Slovenia. Although a significant share of software projects were small or micro-sized, future studies including smaller companies (e.g., SMEs) would provide further valuable insights into the differences between companies varying by size. Third, we did not consider conflict management in our study. Future studies may study the role of conflict management styles and success in software projects. Fourth, we did not control whether users were part of the project team. User involvement by their inclusion in the project team may affect user resistance in a positive way, possibly affecting the role of project team conflicts. In addition to these considerations, future studies may investigate whether it matters which users are involved in the project team (e.g., opinion leaders). Fifth, our study only identified associations between conflicts and user resistance to change. More in-depth insights may be gained with other research designs, such as case study and action research. Sixth, this study investigated only the direct relation between project team conflict and user resistance. Since an indirect causal relation between conflict and resistance is likely, future studies exploring potential mediating factors, such as communication and user involvement would be valuable. Seventh, the study was conducted in 2016. Albeit there were no revolutionary changes in software development from the perspective of conflict and change management since the study was conducted, recent developments in these two fields may lower the ecological validity of this study. A replication study would therefore be useful in strengthening its ecological validity. Eighth, this study was conducted in a single industry. Comparative and cross-industry studies would be beneficial for determining whether the findings are relevant beyond the IT industry.

## Supporting information

S1 Dataset Survey data. (CSV)

S1 Text Legend for survey data. (TXT)

S1 File Survey questionnaire. (PDF)
