# Supplementary material for: The relation between project team conflict and user resistance in software projects
Source: PLoS One. 2021 Nov 16;16(11):e0260059. doi: 10.1371/journal.pone.0260059 (PMC8594807; doi:10.1371/journal.pone.0260059)
Supplement: S1 File — (PDF) [file pone.0260059.s003.pdf]

Note: This is a trimmed version of the survey questionnaire as we do not have permission to publish it in full.

■ Survey on success factors of software solution implementation

All answers should refer to one of the most important software implementation projects that you have completed in the last three years and that you still remember well.

Mark the type of methodology that was used in the selected project:

a Agile methodology (e.g., Scrum, eXtreme Programming)
b Traditional methodology (e.g., Rational Unified Processes, Information Engineering, Custom Development Method)
c We did not use a formal methodology

Mark the type of software solution that was developed within the project:

a New custom software solution
b Customized standard software solution from a local provider (e.g., Marand, Datalab, Adacta)
c Customized standard software solution from an international provider (e.g., SAP, Oracle, Microsoft, IBM)

Number of project team members without external contractors (without outsourcing):

____

Number of external members (outsourced part) of the project team:

____

Total project costs:

a Less than 100,000 €
b Between 100,000 € and 250,000 €
c Between 250,000 € and 500,000 €
d Between 500,000 € and 1,000,000 €
e Between 1,000,000 € and 2,500,000 €
f Between 2,500,000 € and 5,000,000 €
g Between 5,000,000 € and 10,000,000 €
h 10,000,000 € or more

Project duration:

a Less than 3 months
b Between 3 and 6 months
c Between 6 and 12 months
d Between 12 and 18 months
e Between 18 and 24 months
f Between 24 and 30 months
g Between 30 and 36 months
h 36 months or more

Mark your agreement with the statements about what was going on within the project team (circle a number):

	Strongly disagree	Disagree	Somewhat disagree	Neutral	Somewhat agree	Agree	Strongly agree
There has been much relationship tension in the project team.	1	2	3	4	5	6	7
People often got angry while working in the project team.	1	2	3	4	5	6	7
There has been much emotional conflict in the project team.	1	2	3	4	5	6	7
There has been much conflict of ideas in the project team.	1	2	3	4	5	6	7
There have been frequent disagreements within the project team about the task of the project.	1	2	3	4	5	6	7
People in the project team frequently had conflicting opinions about the project.	1	2	3	4	5	6	7
There have been frequent disagreements about who should do what in the project team.	1	2	3	4	5	6	7
There have been many conflicts about task responsibilities in the project team.	1	2	3	4	5	6	7
There have been frequent disagreements about resource allocation in the project team.	1	2	3	4	5	6	7

Mark your agreement with the statements about the response to the project (circle a number):

	Strongly disagree	Disagree	Somewhat disagree	Neutral	Somewhat agree	Agree	Strongly agree
There have been many users resisting the project or the deployed solution.	1	2	3	4	5	6	7
There have been many cases in which user departments did not reply to the request of the project team.	1	2	3	4	5	6	7
